# The effect of long-term alendronic acid treatment on Modic changes in the lumbar spine: a gender and age-matched study

**DOI:** 10.1186/s13018-024-04780-2

**Published:** 2024-05-12

**Authors:** Kenneth Yiu, Hyunjeong Ahn, Graham Ka-Hon Shea

**Affiliations:** Department of Orthopaedics and Traumatology, Li Ka Shing Faculty of Medicine, The University of Hong Kong, Pokfulam, Hong Kong

**Keywords:** Modic changes, Lumbar spine, Osteoporosis, Alendronic acid, Low back pain, Bisphosphonate

## Abstract

**Background:**

Low back pain (LBP) affects a significant proportion of the adult population. Potent anti-resorptive drugs such as intravenous zoledronic acid have been demonstrated to reduce Modic changes (MCs) upon magnetic resonance imaging (MRI) of the spine and concomitantly decrease associated LBP. It is uncertain whether oral alendronic acid has a similar effect.

**Methods:**

82 subjects were recruited in this case-control study. Treatment subjects (*n* = 41) received oral alendronic acid treatment for at least 1-year and were matched by gender and age (± 2) to control subjects (*n* = 41) not receiving any anti-osteoporotic medication. The prevalence, type, and extent of MCs were quantified upon T1 and T2-weighted MRIs of the lumbosacral spine.

**Results:**

Treatment subjects received oral alendronic acid for 124.0 ± 62.1 weeks at the time of MRI assessment and exhibited a lower prevalence of MCs over the lumbosacral spine (18/41 vs. 30/41, *p* < 0.001) as compared to control subjects. Amongst both groups, type 2 MCs were predominant. Quantification of type 2 MCs in treatment subjects revealed a significant reduction in area (113 ± 106 mm^2^ vs. 231 ± 144 mm^2^, *p* < 0.01) and volume (453 ± 427 mm^3^ vs. 925 ± 575 mm^3^, *p* < 0.01) affected by type 2 MCs in comparison to matched controls.

**Conclusion:**

Oral alendronic acid may be useful in the treatment of MC-associated LBP in patients with concomitant osteoporosis.

## Background

Up to 80% of people experience low back pain (LBP) at some point in their lifetime [1], which can cause notable physical and psychological impairment. This results in substantial direct and indirect expenses for the healthcare industry and the economy as a whole [2]. According to a study conducted between 1990 and 2019, the global burden of LBP resulted in 63.7 million disability-adjusted life years [3].

Over 90% of LBP cases are considered non-specific, making it difficult to determine the exact cause of the pain, thereby posing a challenge for effective management [4]. In recent decades we have discovered that radiological alterations in the vertebral endplate and surrounding bone marrow, visible on MRI, are linked to LBP [5, 6]. These changes, known as Modic changes (MCs), were first described by Michael Modic [7] and are classified into three types based on T1 and T2-weighted signal intensity. They are most commonly found at the L4/5 and L5/S1 vertebral levels [8]. The correlation between these specific radiological changes and LBP has led to the identification of a subgroup of patients with LBP who may be treated with therapeutic agents that target bony metabolism.

Previous studies have demonstrated that potent anti-resorptive drugs such as intravenous zoledronic acid reduce LBP in patients with MCs [9]. A decrease in pain scores was associated with a conversion of MC type, as well as decrease in area and volume affected by MCs. Towards ease of administration, a recent clinical trial administered oral zoledronic acid and found a similar reduction in both MCs as well as LBP [10]. However, this study drug did not proceed towards commercialization. Alendronic acid is approximately 20-fold less potent than zoledronic acid [11] and ingested orally as either a daily (10 mg) or weekly (70 mg) regimen that is recommended as a cost-effective first-line treatment option for post-menopausal osteoporosis [12]. Continuous treatment is advocated for up to 5 years, after which a drug holiday is recommended in view of risks of cumulative bisphosphonate exposure. The use of such nitrogen-containing oral bisphosphonates of milder potency is commonplace in the treatment of osteoporosis and has long been reported to reduce back pain [13, 14]. Nevertheless, it remains unknown how treatment affects MCs. Here, we characterized MCs in patients receiving long-term oral alendronic acid in comparison to matched controls not receiving anti-resorptive drugs.

## Methods

### Subject recruitment

In this cross-sectional case-control study we performed a search upon the Clinical Data Analysis and Reporting System (CDARS) database [15] for subjects aged 60 years and over who had received magnetic resonance imaging (MRI) of the lumbosacral spine at Queen Mary Hospital between 1/1/2016 and 31/12/2020 and were concomitantly taking alendronic acid 70 mg once a week for the management of osteoporosis (treatment) or were not receiving any anti-osteoporotic medication (control). CDARS is an electronic database managed by the Hong Kong Hospital Authority, which is a public healthcare statuary body managing 43 public hospitals and institutions, as well as 122 out-patient clinics. Data from the participating institutions are automatically uploaded to the CDARS system for reporting, auditing, and research purposes [16]. The indication for an MRI was most often back pain associated with lower limb sensory or motor disturbances. For the treatment group, we ensured intake of oral alendronic acid for at least 1-year. For the control group, we excluded patients who had previously received any anti-osteoporotic drugs, including those undergoing a drug holiday. From the combined cohort, we excluded patients with L4, L5, or S1 vertebral collapse, spinal surgeries affecting L4-S1, those with prior / active spinal infection or malignancy, and subjects with ankylosing spondylitis since these affected MRI signal intensities. Subjects within the treatment group were matched by means of gender and age (± 2 years) to control subjects.

### MRI assessment of Modic changes

**Fig. 1:**
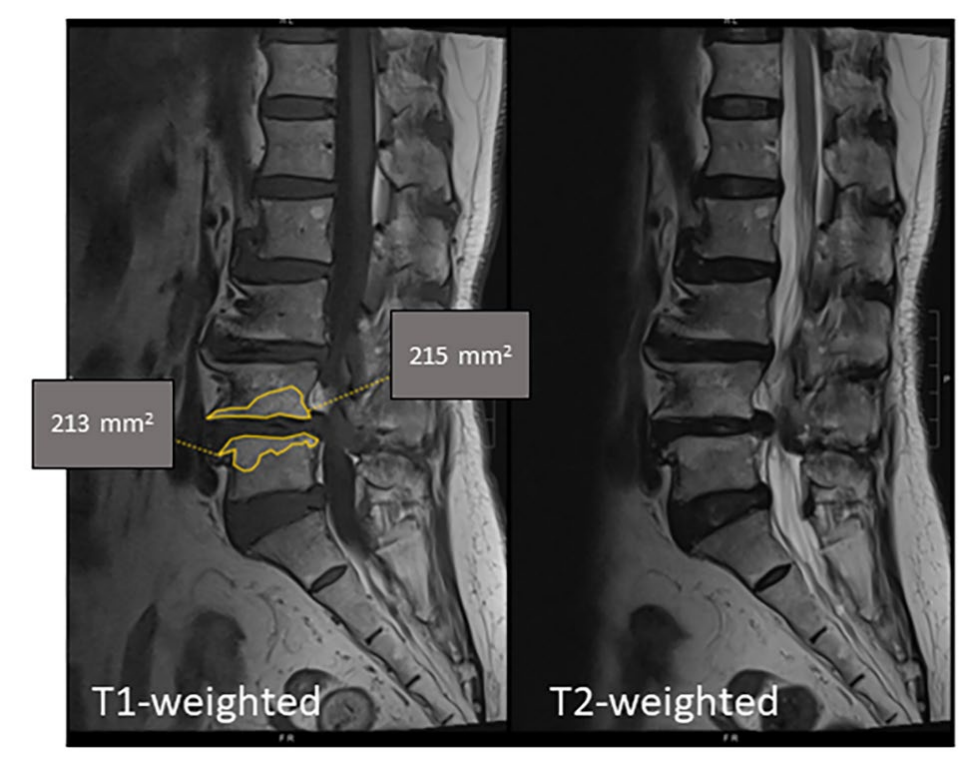
Measurement of Modic changes. T1 and T2-weighted lumbosacral spine MRI sequences in a patient with Type 2 Modic changes over the L4 and L5 vertebra. The area with Type 2 changes is demarcated in yellow. Grading of the affected area in L4 corresponds to ‘C’ since > 50% of the vertebral height is affected. Volume of MC was calculated over the worst affected vertebral level by multiplying the area of MC by the slice thickness of 4 mm

The MRI examinations at our hospital were performed largely using a 1.5T scanner (Siemens, Munich, Germany or Philips, Best, The Netherlands) [17]. T1 and T2 lumbosacral MRI sequences of subjects fulfilling the recruitment criteria were assessed for MCs. We only evaluated L4 – S1 levels, as these levels were most prevalent for MCs [18] and less likely to be affected by osteoporotic fractures which tend to occur around the thoracolumbar junction / upper lumbar levels [19]. Two researchers assessed for the presence / absence of MCs, the type of primary MC change [1–3], and the grading based on the extent of vertical involvement over the most-affected sagittal cut (A = < 25% involvement; B = 25–50% involvement; C = > 50% involvement) [20]. The primary MC was defined in accordance with the type most likely to be associated with LBP [17]. A representative MRI with measurement of type 2 MC area is shown in Fig. 1. The volume of MC was also calculated over the worst affected vertebral level by multiplying the area of MC by the slice thickness of 4 mm. The primary outcome of the study was the prevalence of MCs amongst the treatment group in comparison to controls. Secondary outcomes included the area (mm^2^) and volume (mm^3^) affected by MCs.

### Statistical analysis

A total of 82 subjects were recruited, with 41 in the treatment group matched to 41 in the control group. In addition to the radiographic parameters described above, details of demographic variables and alendronic acid treatment duration were retrieved. Categorical values (i.e. MC type) were compared by chi-squared test, whilst continuous variables with a normal distribution (i.e. MC area and volume) were compared by paired T-testing. Statistical significance was established via two-tailed testing upon a *p*-value < 0.05. Post-hoc one-tailed analysis demonstrated excellent statistical power with the given sample size (0.987). Statistical analysis was carried out on Excel 2021. Power analysis was performed with G*Power software suite [21].

## Results

### Patient recruitment

**Fig. 2:**
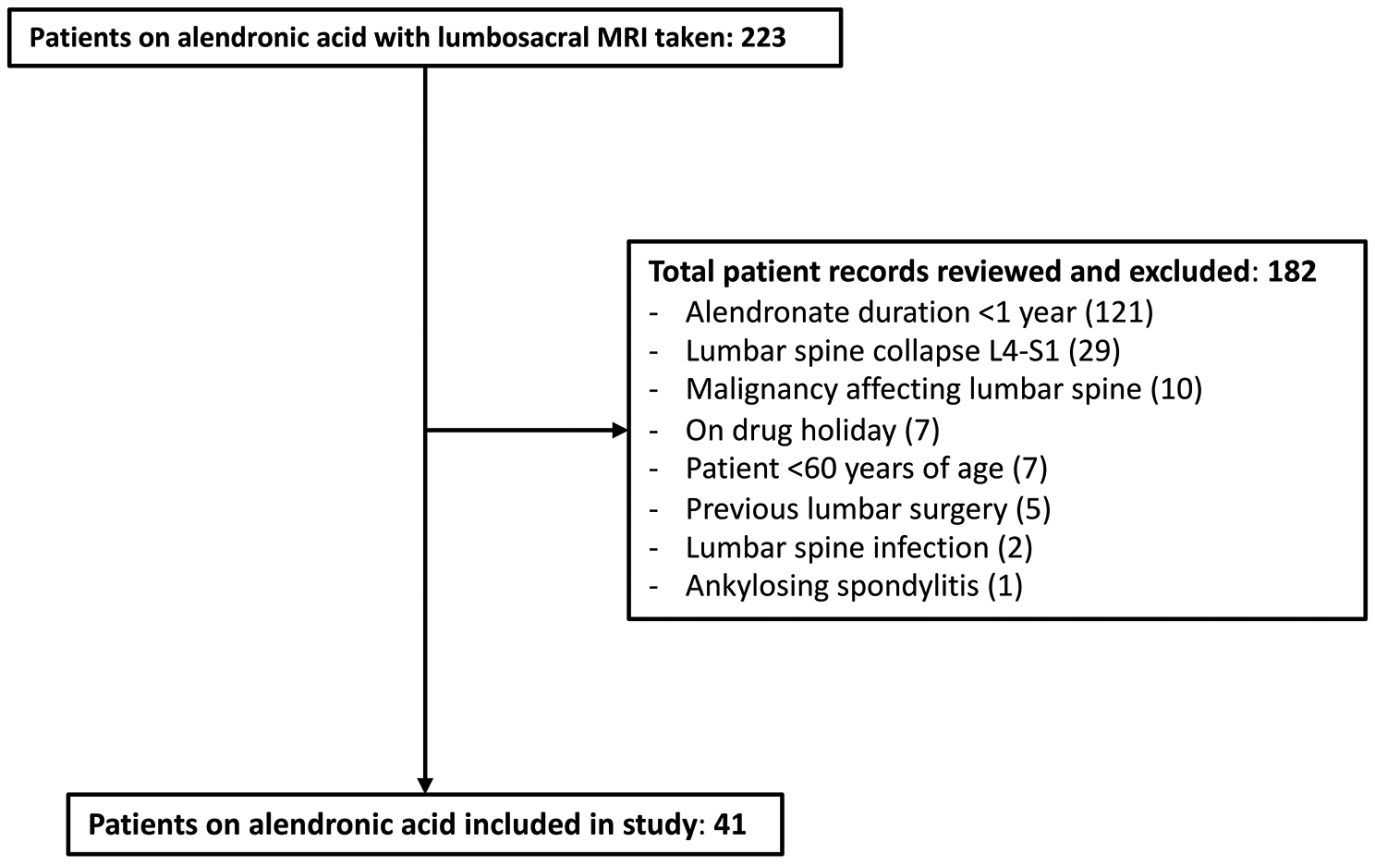
Subject recruitment flow chart

We identified 41 treatment subjects from 223 patients receiving a lumbosacral MRI from 2016 to 2020 (Fig. 2). Amongst the 182 patients excluded patients, 121 received an insufficient duration of alendronic acid, 29 possessed lumbar spine collapse at the level of MC involvement, 10 had concomitant malignancy affecting the lumbar spine, 7 were undergoing a break in treatment (drug holiday or non-compliance), 7 were aged < 60 years old, 5 had previous lumbar surgery at the level of MC involvement, 2 had lumbar spine infection at the level of MC involvement and 1 had ankylosing spondylitis. Treatment subjects were matched by gender and age (± 2) to 41 control subjects receiving a lumbar MRI within the same period who were not on bisphosphonates with details shown on Table 1. The average duration of bisphosphonate use in the treatment group was 124.0 ± 64.1 weeks.

**Table 1:** Subject demographics and description of Modic changes

	Control (*n* = 41)	Treatment (*n* = 41)	*P*-value
Mean age at exam	73.8 ± 7.3	74.1 ± 7.3	0.881
Gender (F/M)	37/4	37/4	1.0
Alendronic acid treatment duration (weeks)	NA	124.0 ± 64.1	NA
Modic Changes: Yes / No	34 / 7	21 / 20	**< 0.001**
- Modic type			
- Type 1 - Type 2 - Type 3	2 31 1	1 19 1	0.0763
- Modic grade			
- A - B - C	22 11 1	19 2 0	**0.0124**
- Level of involvement			
- L4/5 - L5/S1	18 16	10 11	**0.0237**

### Prevalence, type, and grading of Modic changes

The overall prevalence of MCs was significantly higher amongst the control group (Table 1), being present in 34/41 subjects compared to 21/41 subjects in the treatment group (*p* < 0.001). This corresponded to an odds ratio of 0.35 (0.134–0.917, *p* = 0.033). MCs were predominantly type 2, accounting for 31/34 and 19/21 within each respective group. There were no patients with mixed type MCs. MCs were more extensive in the control group as compared to treatment group, with B and C grading according to affected vertebral height in 12/34 treatment subjects as compared to 2/21 control subjects (*p* = 0.012). The level of involvement for MCs was predominantly over the L4 and L5 vertebra in the control group, as opposed to L5 and S1 vertebra in the treatment group (*p* = 0.024).

### Quantification of type 2 Modic changes

Type 2 MCs were predominant amongst the combined cohort and characterized further. The prevalence of type 2 changes was significantly higher (*p* < 0.01) in the control group (31/41, 75.6%) as compared to the treatment group (19/41, 46.3%). We excluded from subsequent area and volume calculations one matched treatment / control pair which had discordant MCs (type 3 in treatment, type 2 in control). The average area of type 2 changes among the treatment group (113 ± 106 mm^2^) was significantly lower (*p* < 0.01) than that of the control (231 ± 144 mm^2^). Similarly, the average volume of type 2 changes in the treatment group (453 ± 427 mm^3^) was significantly lower (*p* < 0.01) than that of the control group (925 ± 575 mm^3^). These results are summarized in Table 2. Subgroup analysis was performed amongst the treatment group with relation to duration of alendronic acid intake and MCs. No significant difference in average area or volume of type 2 MCs was detected between patients receiving within 2 years, or 2 or more years of alendronic acid treatment (*p* = 0.834).

**Table 2:** Quantification of Type 2 Modic changes

	Control (*n* = 41)	Treatment (*n* = 41)	*P*-value
Number with type 2 changes* - Age at exam - Gender (F: M)	30 / 41 72.4 ± 6.8 27:3	18 / 41 73.2 ± 5.6 17:1	**< 0.001** 0.973 1.0
Area with type 2 change (mm^2^)			
- Overall - ≤ 2 years of treatment - > 2 years treatment	231 ± 144	113 ± 106 107 ± 101 118 ± 115	**< 0.01** 0.834
Volume with type 2 change (mm^3^)			
- Overall - ≤ 2 years of treatment - > 2 years treatment	925 ± 575	453 ± 427 427 ± 403 470 ± 460	**< 0.01** 0.834

*One matched pair excluded from analysis were excluded from analysis as MC type was mismatched (type 2 in treatment subject, type 3 in control subject)

## Discussion

In this case control study, patients receiving oral alendronic acid treatment for at least 1-year demonstrated a lower prevalence of MCs over the lumbar spine. Treatment also resulted in a reduced area and volume of type 2 MCs in comparison to matched controls. Our results reveal that MCs, and potentially LBP, may be attenuated by oral alendronic acid intake.

Modic changes are proposed to occur following damage to the disc and endplate which results in an immune response from adjacent bone marrow [22]. Nitrogen-containing bisphosphonate such as alendronic acid reduce bony resorption by osteoclasts via selective inhibition of farnesyl pyrophosphate synthase [11]. Prior studies investigating the effects of anti-resorption drugs on MCs have favoured more potent formulations such as oral and intravenous zolendronic acid [10, 23]. Denosumab given subcutaneously has also been found to have a comparable result to IV zoledronic acid in treating MC-associated LBP at 6-months, whist being associated with less adverse effects [9]. Alendronic acid intake of 6-months has been reported to improve pain scores and quality of life measures in postmenopausal osteoporotic females [13] which was associated with a reduction in biochemical markers of bony turnover. Remarkably, the reduction of pain in accordance with facial scale score (FSS) was 55% at 6-months post treatment with weekly oral alendronic acid as compared to weekly elcatonin injection, with the FSS declining from 6.1/10 to 2.7/10 in the alendronic acid group [13], and others have similarly reported significant reductions in limited-activity and bed-disability days following the use of oral alendronic acid [14]. As a pathophysiological correlate, a post-menopausal animal model receiving alendronic acid demonstrated reduced degenerative changes in the nucleus pulposus and annulus fibrosis [24]. Here, we describe for the first time a radiological difference in MCs in patients receiving long-term alendronic acid treatment in comparison to matched controls not on bisphosphonates, which may be an underlying mechanism by which LBP and functional measures improved in related clinical trials [13, 14].

Our study found type 2 MCs to predominate amongst the cohort. Others have similarly reported on the abundance of type 2 changes amongst the general population [25] as well as patient subgroup attending the spine clinic [10], and an increased prevalence has been reported with aging [26]. The association between MC and LBP is clearest with type 1 changes [8, 27] which are thought to represent bony edema and inflammation [28]. Conversion of type 1 changes to type 2 or type 3 changes is well-correlated with a decrease in LBP. Although fewer in number, prior studies have also demonstrated a correlation between a reduction in type 2 MCs and LBP [27, 29]. Additionally, a recent histological study has indicated type 2 MCs to represent fibroinflammatory changes with complement system activation, features consistent with a pain generator. Regardless of MC type, the extent of reduction in MC area and volume compared favourably to studies utilizing more potent anti-osteoporotic medications [9, 10, 30]. In combination with prior findings on the effect of oral alendronic acid on improving LBP and function, our findings suggest that oral bisphosphonates should be prescribed in osteoporotic patients not only to improve bony turnover, but to alleviate LBP especially in patients with pre-existing MCs.

Limitations to the study include the cross-sectional study design whereupon we were unable to establish a temporal link between alendronic acid intake and conversion in MC type and reduction of MC area / volume since there were no serial MRIs for comparison, nor of routinely available MRIs prior to the initiation of bisphosphonate treatment as compared to after. Another important omission from our study was patient reported outcome measures and functional scores, as these were not routinely assessed at the time of imaging. There was also significant heterogeneity in the dosing period of alendronic acid, and recruitment of larger sample sizes may have enabled for better subgroup analysis of dosing duration in relation to MCs, which this study found to be insignificant.

## Conclusions

Our study indicated that oral alendronic acid intake was associated with reduced MCs. This provided a radiological correlate to explain prior findings on the efficacy of alendronic acid in relieving back pain in the osteoporotic population. Upon further investigation, oral alendronic acid may be a treatment option for MC-associated back pain that is inexpensive and easily administrated as compared to more potent anti-resorption drugs.

## Data Availability

No datasets were generated or analysed during the current study.
